# Enhancing antibiotic therapy through comprehensive pharmacokinetic/pharmacodynamic principles

**DOI:** 10.3389/fcimb.2025.1521091

**Published:** 2025-02-25

**Authors:** Mohammad Sina Alikhani, Mohsen Nazari, Shima Hatamkhani

**Affiliations:** ^1^ Student Research Committee, Urmia University of Medical Sciences, Urmia, Iran; ^2^ Department of Microbiology, Hamadan University of Medical Sciences, Hamadan, Iran; ^3^ Infectious Disease Research Center, Avicenna Institute of Clinical Sciences, Avicenna Health Research Institute, Hamadan University of Medical Sciences, Hamadan, Iran; ^4^ Department of Clinical Pharmacy, School of Pharmacy, Urmia University of Medical Sciences, Urmia, Iran

**Keywords:** pharmacokinetic, pharmacodynamic, antibiotics, antimicrobial efficacy, post-antibiotic effect

## Abstract

Antibiotic therapy relies on understanding both pharmacokinetics (PK) and pharmacodynamics (PD), which respectively address drug absorption, distribution, and elimination, and the relationship between drug concentration and antimicrobial efficacy. This review synthesizes decades of research, drawing from *in-vitro* studies, *in-vivo* models, and clinical observations, to elucidate the temporal dynamics of antibiotic activity. We explore how these dynamics, including concentration-effect relationships and post antibiotic effects, inform the classification of antibiotics based on their PD profiles. Additionally, we discuss the pivotal role of PK/PD principles in determining optimal dosage regimens. By providing a comprehensive overview of PK/PD principles in antibiotic therapy, this review aims to enhance understanding and improve treatment outcomes in clinical practice.

## Introduction

Pharmacokinetics (PK) examines the absorption, distribution, metabolism, and excretion of drugs within the body, while pharmacodynamics (PD) investigates the physiological and biochemical effects of drugs on living organisms, including their mechanisms of action. In essence, PK studies how the body processes drugs, whereas PD focuses on the resultant biological effects these drugs produce (Mittal et al., 2023; Zakaraya et al., 2024).

In the context of antibiotics, PK explores the time-dependent dynamics of drug concentration at infection sites, while PD explains the intricate relationship between antibiotic concentration and antibacterial efficacy (Rao and Landersdorfer, 2021; Sun et al., 2022). Over the past few decades, extensive research has emphasized the variability in antibacterial activity over time among different classes of antibiotics (Medvedeva et al., 2023). Understanding the temporal patterns of antibacterial effects is crucial in determining optimal dosing regimens and therapeutic strategies (Rodríguez-Gascón et al., 2021; Shiva et al., 2022).

This comprehensive review aims to clarify the fundamental principles of PK and PD, particularly regarding antibacterial agents. Additionally, we will explore various metrics used to assess the antibacterial potency of antibiotics and discuss their application in optimizing the clinical use of these agents. Through this examination, we seek to offer insights into improving the efficacy and precision of antibiotic therapy. To achieve this, we conducted a literature search using PubMed, Google Scholar, and Web of Science, targeting studies related on antibiotic PK and PD, including keywords such as “antibiotic efficacy,” “PK/PD relationships,” “time-kill studies,” “post antibiotic,” and “antibacterial resistance.”

## Antibiotic pharmacodynamics

Assessing the antibacterial efficacy of an antibiotic involves several key factors. Firstly, the antibiotic must efficiently reach the site of infection, penetrate the cell wall of the pathogen, and maintain a sufficiently high concentration to exert its bactericidal or bacteriostatic effect. For bactericidal antibiotics, this process requires precise targeting of specific mechanisms within the bacterial cell, leading to inhibition of vital processes crucial for bacterial survival (Baran et al., 2023). In contrast, bacteriostatic antibiotics inhibit the growth and reproduction of bacteria without necessarily killing them, thereby allowing the immune system to eliminate the infection (Halawa et al., 2024). Both types of antibiotics must effectively reach the infection site and sustain appropriate concentrations to be effective (Buonavoglia et al., 2021).

Antibacterial activity serves as a crucial metric, indicating the ability of an antibiotic to inhibit bacterial growth. It is quantified through various indicators, each reflecting different aspects of the antibiotic’s potency against bacteria (Yan et al., 2021). These indicators, such as minimal inhibitory concentration (MIC), minimum bactericidal concentration (MBC), time-kill studies, post antibiotic effect (PAE), post antibiotic effect-sub MIC effect (PAE-SME), and post antibiotic leukocyte effect (PALE), provide valuable insights into an antibiotic’s effectiveness in combating bacterial infections and guiding therapeutic interventions.

### Minimal inhibitory concentration

The MIC is a critical measure of an antibiotic’s antibacterial activity, representing the lowest concentration required to inhibit the growth of microorganisms in *in-vitro* sensitivity tests (Nazari et al., 2024b). Pathogens are typically considered sensitive to an antibiotic when serum concentrations reach at least four times the MIC following the standard dose (Andrews, 2001; Frimodt-Møller, 2002). However, a low MIC does not always correlate with clinical efficacy, as various factors affect the distribution and effectiveness of antibiotics within the body. Even if the serum concentration surpasses the MIC, a significant portion of the antibiotic may remain bound to serum proteins, limiting its ability to diffuse into tissues. This renders the MIC less accurate in predicting antibacterial activity at specific infection sites, such as the cerebrospinal fluid, prostate, or abscesses, while other biological fluids, like urine or bile, may present antibiotic concentrations that far exceed the MIC, ensuring more effective antibacterial action (van Os and Zeitlinger, 2021; Cruz-López et al., 2022).

Furthermore, MIC values derived from *in-vitro* testing often fail to predict *in-vivo* responses, as environmental factors at the infection site (e.g., acidity, oxygen levels), pathogen load, and the emergence of resistant strains can significantly influence antibiotic efficacy (Quek et al., 2022). The inoculum effect, where high pathogen loads reduce antibiotic sensitivity, and the Eagle effect, where higher antibiotic concentrations paradoxically promote pathogen survival, add further complexity to MIC interpretation (Prasetyoputri et al., 2019; Ngo et al., 2021). Additionally, MIC testing in artificial media does not account for the temporal dynamics of antibiotic concentrations, limiting its ability to assess the persistence of inhibitory effects or the optimal dosing regimens necessary for sustained antibacterial activity. The static nature of MIC testing overlooks the changes in antibiotic levels over time, reducing its effectiveness in evaluating continuous antibacterial action once concentrations fall below the MIC threshold (Landersdorfer and Nation, 2021).

Despite its limitations, MIC remains widely utilized due to its simplicity, high reproducibility, and ability to approximate the efficacy of free antibacterial agents at infection sites (Dafale et al., 2016). However, interpreting MIC results requires a detailed understanding of how antibiotic PK, bacterial physiology, and host factors interact to guide effective treatment strategies in clinical practice.

### Minimum bactericidal concentration

MBC is defined as the lowest concentration of antibiotics capable of reducing the pathogen count by at least 99.9%, typically from 10^5^-10^6/^mL to ≤10^2^-10^3/^mL (Nazari et al., 2024a). Most bactericidal antibiotics reduce bacterial counts by over 99.9% even at MIC concentrations, so MIC and MBC values often align closely (Rodríguez-Melcón et al., 2021). However, MBC serves as a crucial indicator in scenarios demanding potent sterilizing effects to eradicate causative bacteria, especially in patients with compromised host defense mechanisms, such as those with meningitis, endocarditis, or neutropenia (Tashmukhambetov, 2016).

In some cases, the MBC may exceed the MIC significantly, a phenomenon known as tolerance, which is applicable only to bactericidal antibiotics (Liu et al., 2020). Tolerance occurs when the MBC/MIC ratio reaches 32 or higher, resulting in reduced bactericidal efficacy and a shift towards a bacteriostatic effect or a gradual decline in bacterial viability (Woods and Washington, 1995; Murray, 2015). However, it is important to note that there’s currently no evidence suggesting that strains exhibiting tolerance elicit poorer treatment responses or prognosis.

### Time-kill study

A time-kill study is a method used to evaluate the temporal dynamics of antibacterial activity by assessing how sterilization levels change over time following antibiotic administration (Garvey, 2023; Nazari et al., 2024c). Unlike static methods such as MIC or MBC, which measure bacterial inhibition or eradication after a fixed period of overnight exposure to a constant antibiotic concentration, the time-kill study provides a dynamic perspective by tracking bacterial count reduction over varying time intervals (Balouiri et al., 2016).

In addition to traditional time-kill studies, *in vitro* PK/PD models, such as the one-compartment model and the hollow fiber infection model (HFIM), are also valuable tools for assessing antibiotic efficacy (Sadouki et al., 2021). The one-compartment model simulates the distribution and elimination of an antibiotic in a single compartment, providing insights into how the drug behaves over time within a controlled environment (Kristoffersson, 2015). The HFIM, which more closely mimics *in vivo* conditions, consists of a hollow fiber system through which antibiotic is continuously perfused, allowing for the study of bacterial growth and antibiotic exposure over time (Kembou-Ringert et al., 2023). This model is particularly useful for simulating infection conditions in humans, making it a powerful tool for investigating bacterial responses to antibiotic treatment (Ferro et al., 2015). This method is frequently employed to ascertain whether synergistic effects exist when antibiotics are used in combination (Acar, 2000).

Despite its utility, the time-kill study has limitations. Administering antibiotics at a constant concentration does not accurately replicate the natural fluctuation of antibacterial agent levels within the human body (Levison and Levison, 2009; Nazari et al., 2025). Moreover, the study environment typically lacks the continuous provision of nutrients required for pathogen growth, thereby skewing the results towards antibacterial activity (Storflor, 2024). Additionally, such studies overlook variables, such as metabolites, which may influence the antibacterial efficacy of antibiotics *in-vivo* (Teo et al., 2021).

### Post antibiotic effect

The post-antibiotic effect (PAE) is the phenomenon where bacterial growth remains suppressed even after the removal of an antibiotic, indicating a temporary cessation of microbial activity (Proma et al., 2020). PAE can occur with both bactericidal and bacteriostatic antibiotics, though it is more pronounced with bactericidal agents. This period of suppressed growth is defined by the time required for microorganisms to resume normal metabolic functions (Pai et al., 2015). PAEs are primarily observed with certain classes of antibiotics, including aminoglycosides, fluoroquinolones, tetracyclines, clindamycin, and rifampin, which target essential cellular processes such as protein and nucleic acid synthesis (Duong et al., 2021; Apley, 2022; Himstedt et al., 2022). Notably, PAEs are especially pronounced for antibiotics that inhibit protein and nucleic acid synthesis (Li et al., 2022).

PAE can affect both bacteria that survived the antibiotic treatment and those that are newly infecting. In most studies, these effects are observed under *in-vitro* conditions, though PAE can also occur *in-vivo* under specific circumstances. The manifestation of PAE can differ depending on the mechanism of action of the antibiotic, the bacterial species, and whether the antibiotic is bactericidal or bacteriostatic. For instance, β-lactam and glycopeptide antibiotics, which target the bacterial cell wall, typically exhibit PAE in gram-positive bacteria but show minimal or no PAE in gram-negative bacteria (Dörr, 2021; Baran et al., 2023). However, carbapenem antibiotics are an exception, as they can induce PAE even in gram-negative bacteria, including *Pseudomonas aeruginosa* (Fuste et al., 2013).

The *in-vivo* applicability of PAE is more complex, as laboratory conditions do not always replicate the body’s dynamic antibiotic levels or immune responses. For example, when *Streptococcus pneumoniae* is exposed to penicillin and cephalosporin, PAE is observable *in-vitro* but remains undetectable *in-vivo* (Majcherczyk et al., 1994; Kaldalu et al., 2020). *In-vivo* studies have also shown discrepancies in the persistence of aminoglycoside PAE when administered at prolonged intervals or with repeated dosing (Salehifar and Rafati, 2015). These differences highlight the complex interplay between antibiotic PK, bacterial physiology, and the host’s immune system in determining the duration and magnitude of PAE (van Gent et al., 2021; Chen, 2023).

### Postantibiotic effect-sub MIC effect

At concentrations below the MIC, antibacterial agents exhibit a dual action: slowing bacterial growth and inducing temporary morphological changes, thereby extending the duration of the PAE (Hanberger, 1992). These morphological changes are not due to mutations but are reversible alterations in bacterial structure that impair viability without permanently altering the bacterial genome. Notably, the combined PAE-SME is observed to persist longer *in-vivo* compared to *in-vitro* settings (Saravolatz et al., 2017; Baquero and Levin, 2021). This persistence highlights the significant role that physiological factors play in antibiotic efficacy within a living organism (Grant and Hung, 2013). *In-vivo* conditions, such as the presence of immune system components, tissue-specific factors, and varying biochemical environments, contribute to the sustained antibacterial activity observed (Shi et al., 2019). These factors can enhance the duration and effectiveness of the antibiotic’s action, making the *in-vivo* setting more complex and often more favorable for prolonged antibiotic effects compared to the controlled and simplified conditions of *in-vitro* experiments (MacKenzie and Gould, 1993). This extended PAE-SME *in-vivo* underscores the necessity of considering the body’s natural defenses and the overall physiological context when evaluating the true efficacy of antibacterial agents in clinical scenarios (Muteeb et al., 2023).

### Post antibiotic leukocyte effect

Following exposure to antibacterial agents, the PAE can be further prolonged due to the increased susceptibility of bacteria to intracellular phagocytosis or the bactericidal activity of leukocytes (Horgen et al., 1998). This phenomenon, known as the post-antibiotic leukocyte effect (PALE), underscores the importance of host immune cells in enhancing the antimicrobial action of antibiotics. Specifically, the presence of neutrophils has been shown to significantly prolong the PAE of antibiotics such as aminoglycosides and fluoroquinolones against Gram-negative bacteria, often doubling the duration of the effect (Yaneja and Kaur, 2016).

The interaction between antibiotic treatment and the host immune system is complex, with leukocytes playing a crucial role in clearing bacteria during and after antibiotic exposure (Willing et al., 2011). The bactericidal activity of neutrophils, in particular, is enhanced in the post-antibiotic period, further reducing bacterial viability. However, the impact of PALE varies depending on the antibiotic class and the bacterial target (Wu et al., 2024). For instance, in the case of Gram-negative bacteria exposed to β-lactams, the presence or absence of leukocytes does not significantly affect the duration of the PAE. This suggests that the prolonged effect seen with other antibiotics may be due to the unique interactions between those agents and host immune cells (Wu et al., 2024).

## Antibiotic pharmacokinetics

PK deals with the processes governing absorption, distribution, metabolism, and excretion (Balhara et al., 2022). It quantitatively elucidates the dynamic relationship between doses, effects and drug concentration in various body fluids (Mager, 2006). By using PK indicators, clinicians can tailor antibiotic dosing regimens to achieve optimal efficacy while minimizing side effects (Dhole et al., 2023). For example, changes in kinetic parameters can have significant clinical implications, such as in the once-daily administration of aminoglycosides. This dosing strategy takes advantage of the concentration-dependent killing and prolonged PAE of aminoglycosides, allowing for effective bacterial eradication with reduced toxicity. By administering a higher dose once daily, peak serum concentrations are maximized, enhancing bacterial killing, while the extended dosing interval reduces the risk of nephrotoxicity and ototoxicity, common side effects associated with aminoglycoside therapy (Craig, 1995; Eyler and Mueller, 2010).

The concentration of antibiotics in the body typically includes an interstitial and an intracellular compartment, with distribution patterns varying significantly depending on the characteristics of the antibiotic. For example, β-lactam antibiotics often show high concentrations in the interstitial compartment, closely mirroring serum levels, while intracellular concentrations remain low (Barreto et al., 2021). Conversely, fluoroquinolones exhibit low interstitial and serum concentrations, but accumulate significantly in intracellular compartments (Vergalli et al., 2020). Furthermore, distribution dynamics are influenced by bacterial characteristics, as most infections occur within tissues where bacteria predominantly exist extracellularly (Deatherage and Cookson, 2012). Thus, monitoring antibiotic concentrations in the interstitial fluid at the site of infection is a critical determinant of therapeutic efficacy (Abraham et al., 2019).

Serum concentration has been used as a primary indicator, albeit with limitations, as it may not accurately reflect interstitial concentrations. Recent advancements, such as microdialysis techniques, offer promising avenues for directly measuring antibiotic concentrations in the interstitium, providing more precise insights into therapeutic efficacy (Tincu et al., 2023). Microdialysis involves inserting a small probe with a semipermeable membrane into the tissue of interest (de Lange et al., 2000). This probe allows for the continuous sampling of extracellular fluid, providing a real-time measure of the drug concentration directly at the site of infection, thus offering a more accurate assessment of its therapeutic effect (Matzneller and Brunner, 2011).

The protein-binding ability of antibiotics in serum represents another critical PK factor impacting antibacterial activity. Laboratory measurements typically include both the bound and free forms of antibiotics in plasma, yet only the free fraction exerts antibacterial effects at infection sites. Numerous studies have established a direct correlation between the concentration of free antibiotics and their efficacy against specific bacterial strains (Ahmed et al., 2022).

A notable difference in PK between animals and humans is in clearance rates, with smaller animals having faster antibiotic clearance and shorter half-lives than humans. These differences can significantly affect treatment outcomes in animal models of infection (Craig, 2014). However, innovative approaches using uranyl nitrate-induced tubular damage aim to simulate human-like half-lives in animal models, thereby increasing their translational relevance to clinical practice (Beyi et al., 2022).

## Pharmacokinetics/pharmacodynamics relationships

The relationship between PD and PK in antibiotics is crucial for determining effective and safe dosing regimens. PK focuses on the absorption, distribution, metabolism, and excretion of antibiotics, providing insights into how drug concentrations change over time within the body. PD, on the other hand, examines the drug’s biological effects on bacteria, including the mechanisms of action and the relationship between drug concentration and bacterial killing or inhibition. Together, these disciplines help optimize antibiotic therapy by ensuring that drug levels are sufficient to eradicate pathogens while minimizing toxicity and resistance development.

### Pharmacokinetics/pharmacodynamics indicators and antibiotic effectiveness, and classification of antibiotics

Traditionally, early clinical antibiotic dosing was based on achieving serum concentrations above the MIC of the causative bacteria, with dosing intervals loosely informed by serum clearance rates (Tessier and Scheld, 2010). While some trials have compared different concentration levels, there are limited studies investigating the impact of dosing intervals. To identify the most effective indicators for antibiotic success, researchers have utilized animal infection models (Zak and O'Reilly, 1990). Through dose fractionation studies, where antibiotics were administered at different times with the same total dose, dose-effect curves were created to determine reliable predictors of effectiveness (Valero Telleria et al., 2021). For example, a study using a neutropenic mouse pneumonia model evaluated the effectiveness of ceftazidime against *Klebsiella pneumoniae*, revealing that the time (T) maintained above the MIC (T>MIC) was the best predictor of *in-vivo* efficacy (Petraitiene et al., 2020). This finding emphasizes the crucial role of PK/PD indicators in optimizing antibiotic therapy. Through interdisciplinary collaboration and translational research, we can develop tailored treatment regimens that maximize efficacy while minimizing resistance and adverse effects.

The classification of antibiotics based on PK/PD involves categorizing them into three distinct groups according to their concentration-time characteristics and PAE (Minichmayr et al., 2022). PK/PD analysis integrates all relevant data, enabling researchers and physicians to select the most effective antibiotic and dosing regimen for each infection and patient, enhancing antibiotic efficacy while minimizing side effects and reducing the risk of resistance. The three main PK/PD indicators used to predict antimicrobial effects are: the duration the drug concentration exceeds the MIC, the maximum drug concentration to MIC ratio (Cmax/MIC), and the 24-hour ratio of the area under the concentration-time curve to MIC (AUC/MIC) (**Table 1**) (Mouton et al., 2005).

**Table 1:** Categorization of antibiotics according to pharmacokinetic/pharmacodynamic.

Killing pattern and post antibiotic effect	Example antibiotics	Therapy objective and key PK/PD^*^ parameter	Reference
Concentration-dependent with moderate to long-lasting effects	Metronidazole, Aminoglycoside, Daptomycin, Fluoroquinolone, Ketolide	Achieve high serum concentrations; 24-hour AUC/MIC^** ^and peak/MIC	(Drusano et al., 1998; Gibreel and Taylor, 2006; Rybak, 2006; Holfeld et al., 2018; Stübing et al., 2024)
Time-dependent with minimal to no lasting effects	Penicillin, Carbapenem, Cephalosporin, Monobactam	Extend duration of exposure; T>MIC	(Cholewka et al., 1999; Munoz-Price et al., 2016)
Time-dependent with moderate to long-lasting effects	Macrolide, Clindamycin, Glycopeptide, Streptogramin, Oxazolidinone	Optimize maximize amount of drug; 24hr AUC/MIC	(Pea et al., 2004; Paris et al., 2008; Yarlagadda et al., 2016; Kobuchi et al., 2020)

*Pharmacokinetic/Pharmacodynamic

**Area under the concentration-time curve to the MIC

The first category comprises concentration-dependent antibiotics with a long PAE, such as aminoglycosides, fluoroquinolones, ketolides, and daptomycin. These antibiotics exert a faster and more extensive sterilizing effect at higher concentrations (Turnidge, 2003; Quintiliani and Quintiliani, 2008; Levison and Levison, 2009; Heavner et al., 2018). Therefore, administering a high dose helps to maximize their effectiveness. PK/PD indicators such as peak/MIC and AUC/MIC are crucial in predicting the efficacy of these antibiotics (Barger et al., 2003). In contrast, the second category consists of time-dependent antibiotics with low or moderate PAE, such as β-lactams. These antibiotics do not exhibit increased sterilizing power at higher concentrations. Instead, their efficacy depends on prolonged exposure times above the MIC. Therefore, the T>MIC serves as a key indicator for optimizing therapeutic outcomes (Quintiliani and Quintiliani, 2008; Tilanus and Drusano, 2023). Finally, antibiotics in the third category are time-dependent with a long PAE, including azithromycin, tetracyclines, glycopeptides, clindamycin and tetracycline. Although high concentrations of these antibiotics in the body do not increase their sterilizing power, they do have the unique ability to suppress bacterial regrowth for an extended period of time. Therefore, increasing the dose of antibiotics to increase the AUC/MIC ratio is critical to maximize their efficacy (Al Jalali and Zeitlinger, 2018; Tsuji et al., 2018; Haddad et al., 2022; LaPlante et al., 2022).

This comprehensive understanding of antibiotic classification based on PK/PD facilitates tailored therapeutic approaches, ensuring optimal treatment outcomes while minimizing the risk of resistance and adverse effects. Additionally, ongoing research and clinical experience refine our understanding of antibiotic efficacy, guiding advancements in therapy strategies to combat antibiotic resistance. By optimizing dosing regimens to maintain effective drug concentrations and target specific bacterial mechanisms, PK/PD principles help mitigate the development of resistant bacterial strains and improve overall patient care.

### Pharmacokinetics/pharmacodynamics modeling

Determining the right antibiotic dosage can be tricky in clinical trials, but animal models provide a useful setting to determine out the best dosing and PK/PD index values (Wicha et al., 2021). By using non-linear regression and the Hill equation, researchers can analyze dose-response curves to understand the relationships between dose, concentration, and effectiveness. They also experiment with different bacterial strains, including resistant ones, to determine PK/PD index values like static dose, 1-log kill, and 2-log kill dose (Ankomah, 2013).

For β-lactam antibiotics, achieving a 100% T>MIC is not necessary for a significant antibacterial effect (Lenhard and Bulman, 2019). *In-vivo* studies suggest that maintaining serum concentrations above MIC for 30-40% of the dosing interval exhibits bacteriostatic activity (McNabb and Bui, 2001). Conversely, if serum concentrations remain above MIC for less than 20% of the interval, mortality rates approach 100%, while exceeding 40-50% leads to survival rates of 90-100% (Guilhaumou et al., 2019).

The 24-hour AUC to MIC ratio serves as a crucial predictor for fluoroquinolone effectiveness. AUC/MIC values ranging from 25 to 50 correlate with bacteriostatic action in most animal infection models. Mortality rates exceed 50% when the AUC/MIC ratio is below 30 but decline significantly to nearly 0% when the ratio exceeds 100, indicating optimal efficacy (Onufrak et al., 2016).

Various factors influence PK/PD indicators of antibiotics, including administration interval, protein binding rate, bacterial strain or resistance, immune function, site of infection, and initial inoculation concentration (Sy and Derendorf, 2016). For example, β-lactam antibiotics show varying T>MIC values depending on the type of antibiotic, with cephalosporins having a greater value than penicillins, and penicillins having a greater value than carbapenems. This hierarchy is attributed to their differing sterilizing abilities (Turner et al., 2022). Furthermore, the protein binding rate significantly impacts PK/PD index values, with differences observed even within the same antibiotic class (Rao and Landersdorfer, 2021).

PK/PD index values may also vary depending on bacterial strain and host immune status. For instance, the T>MIC requirement is lower in staphylococci compared to Gram-negative rods or streptococci, which is attributed to the presence of *in-vivo* PAE of *staphylococcus* species (Ramos-Martín, 2017). Furthermore, PK/PD index values differ between individuals with normal immune function and those who are neutropenic, particularly for fluoroquinolones, as immune status significantly influences drug efficacy (Scaglione, 2010).

While PK/PD index values generally remain consistent across infection sites, pneumonia presents an exception due to variations in epithelial lining fluid (ELF) penetration (Zhang et al., 2021). For example, vancomycin demonstrates better efficacy in models focusing on non-pneumonia infections due to its limited penetration into ELF (Horn et al., 2017). Conversely, macrolides exhibit enhanced effectiveness in pneumonia models owing to their superior ELF penetration (Davidson, 2019).

## Pharmacokinetics/pharmacodynamics clinical applications

As our understanding of antibiotic PK/PD continues to evolve, its clinical applications span various domains, encompassing both the refinement of existing antibiotic therapies and the development of novel treatment strategies. Here, we delve into the multifaceted applications of PK/PD principles in clinical practice (**Table 2**).

**Table 2:** Clinical applications of pharmacokinetics/pharmacodynamics in antibiotic therapy.

Clinical Application	Details	Reference
Optimizing antibiotic dosing strategies	Studies on antibiotic dosing in animal models highlight the benefits of continuous administration, particularly for β-lactams and fluoroquinolones, with long-half-life antibiotics like ceftriaxone improving therapeutic effects and reducing resistance risks.	(Van Bambeke and Tulkens, 2001; Petri, 2006; Gao et al., 2011; Tasso et al., 2011; Labreche and Frei, 2012; Sharma et al., 2016; Williamson et al., 2017)
Development of new antibiotics	PK/PD^*^ data from animal models guide the development of new antibiotics, ensuring robust clinical efficacy and meeting regulatory requirements for approval.	(Yılmaz and Özcengiz, 2017; Sime and Roberts, 2018; Palmer et al., 2022; Bissantz et al., 2024)
Determination of susceptibility inflection points	PK/PD concepts improve susceptibility testing accuracy across various antibiotic classes, aiding in the selection of effective antibiotics and dosing regimens to combat resistance.	(Seeger et al., 2021)
Application of empirical treatment guidelines	PK/PD principles enhance empirical treatment guidelines across a range of infections, optimizing antibiotic selection and dosing to maximize efficacy and minimize resistance.	(M̈ller et al., 2004; Khalili et al., 2012; Stott and Hope, 2017; Guardabassi et al., 2018; Roggeveen et al., 2020; Marques et al., 2024)
Development of new formulations	PK/PD integration in formulation development uses advanced computational tools to optimize drug exposure, efficacy, and resistance management, considering regional and epidemiological variations.	(Tam and Nikolaou, 2011; Nielsen and Friberg, 2013; Natarajan et al., 2014; Asín-Prieto et al., 2015; Kalhapure et al., 2015; Koo et al., 2017; Organization, 2018; Charumathy et al., 2022; Tang Girdwood et al., 2022; Mishra et al., 2023)

^*^pharmacokinetics/pharmacodynamics

### Optimizing antibiotic dosing strategies

Studies in animal models have examined dosing strategies tailored to the unique PK/PD characteristics of different antibiotic classes. For fluoroquinolones, which exhibit concentration-dependent activity, the peak concentration to MIC (peak/MIC) ratio has been a key focus. Maintaining a peak/MIC ratio of 8-10 or higher has been shown to yield optimal clinical outcomes against Gram-negative bacteria (Tasso et al., 2011; Labreche and Frei, 2012). Similarly, for macrolides and other time-dependent antibiotics, the duration of T>MIC—the time the drug concentration remains above the MIC—has been identified as a critical factor for efficacy (Van Bambeke and Tulkens, 2001).

Comparative experiments have further explored the benefits of intermittent versus continuous dosing regimens, particularly for β-lactams and other antibiotic classes. While intermittent dosing relies on achieving peak concentrations periodically, continuous administration provides a steady antibiotic level, potentially ensuring prolonged T>MIC. Continuous administration has emerged as a favored approach for many antibiotic types due to its ability to improve treatment outcomes and cost-effectiveness (Petri, 2006). However, challenges such as the risk of phlebitis and the potential for resistance development persist with both approaches (Williamson et al., 2017).

To address the challenge of maintaining adequate T>MIC in prolonged treatments, long-half-life antibiotics such as ceftriaxone have shown promise. By sustaining effective drug concentrations in the body over extended periods, these agents help achieve optimal therapeutic effects while minimizing the risk of resistance emergence (Gao et al., 2011; Sharma et al., 2016).

### Development of new antibiotics

PK/PD data obtained from animal infection models serve as a cornerstone in the development of novel antibiotics across various classes, including not only β-lactams but also fluoroquinolones, macrolides, and others. These data provide crucial insights into the PK/PD properties of antibiotics, guiding researchers in identifying predictive indicators of efficacy (Yılmaz and Özcengiz, 2017). By elucidating the concentration-effect relationships and PAE of different antibiotics, preclinical testing enables the formulation of optimized treatment regimens tailored to specific microbial pathogens (Sime and Roberts, 2018).

The integration of PK/PD principles into preclinical studies expedites the translation of new antibiotics from bench to bedside. With comprehensive PK/PD data, researchers can confidently design clinical trials, knowing that the investigational antibiotics demonstrate robust clinical efficacy. Regulatory bodies, such as the U.S. Food and Drug Administration, now mandate the inclusion of PK/PD data as a prerequisite for antibiotic approval (Bissantz et al., 2024). This requirement ensures that new antibiotics undergo rigorous evaluation, guaranteeing their effectiveness and safety in clinical settings (Palmer et al., 2022).

### Determination of susceptibility inflection points

Incorporating PK/PD concepts into susceptibility testing methodologies, as outlined by organizations like the clinical laboratory standards institute, represents a significant advancement in refining susceptibility inflection point determination (Seeger et al., 2021). While the focus often revolves around β-lactam antibiotics, the application of PK/PD principles extends to various antibiotic classes, including fluoroquinolones, tetracyclines, and aminoglycosides. For example, the determination of PD inflection points for fluoroquinolones involves assessing the highest MIC capable of maintaining serum concentrations above a certain threshold for optimal efficacy. This threshold varies depending on the specific antibiotic and the microbial target. By accounting for factors such as drug formulation and the physiological context of infection, susceptibility testing methodologies can more accurately predict antibiotic efficacy in clinical settings (Seeger et al., 2021).

### Application of empirical treatment guidelines

PK/PD principles are fundamental in shaping the application of empirical treatment guidelines for antibiotics, serving as a cornerstone for optimizing therapy while mitigating antimicrobial resistance (Roggeveen et al., 2020). Through the integration of PK/PD considerations, clinicians gain crucial insights into PK behavior of antibiotics, encompassing aspects such as absorption, distribution, metabolism, and excretion (Palmer et al., 2022). Armed with this understanding, they can tailor dosage regimens to ensure optimal drug exposure at the infection site, thereby enhancing therapeutic efficacy (Stott and Hope, 2017).

A key objective of PK/PD-informed treatment guidelines is to maximize therapeutic efficacy by aligning antibiotic dosing with the dynamic interplay between drug exposure and microbial response (Onufrak et al., 2016). This strategy aims to maintain antibiotic concentrations above MIC for an adequate duration, effectively suppressing bacterial growth and eradicating infections.

PK/PD principles are versatile and applicable across various clinical scenarios, encompassing a broad spectrum of infections and patient populations (Marques et al., 2024). Whether managing common infections like otitis media and pneumonia or addressing more complex conditions such as skin and soft tissue infections and urinary tract infections, PK/PD principles guide clinicians in selecting the most appropriate antibiotics and dosing strategies tailored to individual patient needs (M̈ller et al., 2004; Khalili et al., 2012; Guardabassi et al., 2018).

### Development of new formulations

PK/PD integration into the drug development process helps optimize therapeutic efficacy, minimize toxicity, and combat the growing challenge of antimicrobial resistance (Palmer et al., 2022). PK studies determine how the body absorbs, distributes, metabolizes, and excretes a drug. Understanding these processes helps in designing formulations that achieve optimal drug concentrations at the infection site (Organization, 2018). PD studies then relate these concentrations to their antimicrobial effects (Asín-Prieto et al., 2015). Together, PK/PD modeling ensures that new formulations provide sufficient drug exposure to eradicate pathogens while minimizing side effects (Rodríguez-Gascón et al., 2021).

By analyzing the relationship between drug concentration and microbial kill rates (PK/PD indices like Cmax/MIC, AUC/MIC, and T>MIC), researchers can establish effective dosing regimens (Tam and Nikolaou, 2011). This ensures that the new antibiotic formulations deliver the right dose at the right frequency to maintain therapeutic levels and prevent resistance development (Kalhapure et al., 2015). PK/PD principles guide the design of drug delivery systems that enhance the bioavailability and stability of antibiotics (Sy and Derendorf, 2016). Formulations such as sustained-release tablets, liposomal encapsulation, or nanoparticles can be developed to maintain optimal drug levels over extended periods, improving patient compliance and treatment outcomes (Natarajan et al., 2014; Charumathy et al., 2022).

Different infections require different drug concentrations and exposure times. PK/PD studies help tailor new formulations to the specific needs of various infections, such as targeting intracellular bacteria, overcoming biofilm-associated infections, or penetrating difficult-to-reach tissues (Koo et al., 2017; Mishra et al., 2023; Hemmati et al., 2024).

Through the utilization of population PK and microbiological susceptibility data, advanced computational tools like Monte Carlo simulation provide valuable insights into PK profiles of new formulations (Rodríguez-Gascón et al., 2021). By simulating drug exposure in diverse patient populations and accounting for variability in PK parameters, such as clearance and volume of distribution, Monte Carlo simulations enable the prediction of antibiotic concentrations achieved at the site of infection (Nielsen and Friberg, 2013; Tang Girdwood et al., 2022).

## Challenges and limitations

One major challenge is the generalization from animal models to human application due to differences in metabolism, immune responses, and drug clearance rates between smaller animals and humans, which complicates the extrapolation of PK/PD data from animal studies to clinical settings. Additionally, significant discrepancies between *in-vitro* and *in-vivo* environments, such as the presence of immune cells and tissue penetration capabilities, can impact the effectiveness and behavior of antibiotics. *In-vitro*-derived PK/PD models, while valuable for early-stage testing, may not fully replicate the complexities of the *in-vivo* environment, making them less reliable for predicting clinical outcomes.

### PK/PD considerations in special populations

Dosing antibiotics in special populations such as the elderly, obese individuals, and patients with comorbidities presents unique challenges and limitations due to their altered PK and PD (Soraci et al., 2023). In elderly patients, age-related physiological changes impact drug absorption, distribution, metabolism, and excretion, necessitating careful monitoring and dose adjustments (Shi and Klotz, 2011). This is particularly important for drugs like aminoglycosides and vancomycin, where renal function must be closely observed to avoid toxicity (Elyasi et al., 2013; Zamoner et al., 2019).

Obese patients require dosing adjustments based on actual or lean body weight to ensure effective tissue penetration, especially for lipophilic antibiotics such as daptomycin and vancomycin (Payne and Hall, 2014). Similarly, patients with renal or hepatic impairments need tailored dosing strategies to prevent drug accumulation and toxicity (Krens et al., 2019). For instance, beta-lactams and antibiotics like erythromycin and rifampin require specific dose management to maintain therapeutic levels without causing adverse effects (McLawhon, 2012).

Pregnant and lactating women experience physiological changes that alter drug PK, necessitating dose adjustments to ensure both maternal efficacy and minimal drug transfer to breast milk (Pennell, 2003; Anderson, 2006). Pediatric patients, particularly neonates and infants, have different PK profiles compared to adults, requiring age-appropriate dosing strategies to ensure efficacy and avoid toxicity, especially with aminoglycosides (van den Anker and Allegaert, 2019). Practical examples include adjusting vancomycin dosing based on actual body weight in obese patients, aminoglycoside dosing in elderly patients based on renal function, and reducing beta-lactam doses in patients with renal impairment (Dedkaew et al., 2015; Xu et al., 2022; Yoon et al., 2023). These tailored dosing strategies are crucial for optimizing therapeutic efficacy while minimizing toxicity and resistance in these special populations (Sime et al., 2015).

Traditional PK/PD metrics like MIC and MBC offer static snapshots of antibacterial activity but fail to account for dynamic changes in antibiotic concentrations over time (Debanne et al., 2016; Rothery et al., 2024). This limitation hinders their utility in predicting continuous efficacy and optimal dosing regimens in fluctuating clinical environments. Experimental techniques like microdialysis and time-kill studies also have inherent limitations; for instance, time-kill studies do not accurately mimic the decreasing antibiotic levels post-administration *in-vivo* (Zaknoon et al., 2021). Furthermore, the complexity and variability of PAE across different antibiotic classes and bacterial species add to the challenge of consistently predicting the duration and impact of PAE in clinical treatments (Pea et al., 2005).

The variability in PK/PD indicators based on bacterial strain, infection site, and patient immune status complicates the standardization of PK/PD-based dosing strategies, necessitating tailored approaches for different clinical scenarios (Rodríguez-Gascón et al., 2021). This, in turn, can be resource-intensive and require extensive clinical validation. The development of new antibiotics faces significant challenges, including extensive preclinical and clinical testing to ensure efficacy and safety, regulatory requirements for PK/PD data, and the emergence of resistant bacterial strains outpacing the development of new antibiotics. Additionally, integrating PK/PD principles into routine clinical practice requires specialized knowledge, ongoing education and training for clinicians, and the development of accessible computational tools to support decision-making. Addressing these challenges is crucial for advancing antibiotic therapy, necessitating future research to refine PK/PD models and develop robust strategies to overcome obstacles associated with antibiotic resistance and therapeutic optimization.

One of the challenges in investigating antibiotic resistance through PK/PD lies in the expense associated with conducting these tests. Analyzing the intricate interplay between drug exposure, microbial response, and resistance development often requires sophisticated laboratory equipment, specialized expertise, and significant financial resources. From acquiring and maintaining state-of-the-art instrumentation to covering the costs of consumables and personnel training, the financial burden of conducting PK/PD studies can be substantial. This expense can pose a barrier to conducting comprehensive research across diverse antibiotic classes, inhibiting the thorough exploration of resistance mechanisms and hindering the development of effective strategies to combat antibiotic resistance.

## Future perspectives

Looking ahead, the integration of PK/PD principles holds substantial promise for enhancing patient outcomes by tailoring antibiotic therapy to individual needs. By leveraging PK/PD principles, clinicians can design treatment regimens that maximize efficacy while minimizing the risk of resistance development. The continued application of PK/PD principles is poised to be a pivotal strategy in optimizing the use of both existing and newly developed antibiotics. This approach is essential for addressing the urgent need for effective solutions in the fight against infectious diseases.

Future research should focus on further refining PK/PD models and incorporating advanced technologies such as artificial intelligence and machine learning to predict patient-specific responses more accurately. Additionally, interdisciplinary collaboration between pharmacologists, microbiologists, and clinicians will be crucial in translating PK/PD insights into clinical practice. By continuing to innovate and apply PK/PD principles, we can improve therapeutic outcomes, curb the spread of resistance, and ultimately ensure the efficacy of antibiotic treatments for future generations.

## Conclusion

The development of new antibiotics is a challenging and lengthy endeavor, often spanning over a decade. With the escalating threat of antibiotic resistance, an in-depth understanding of PK/PD is crucial for making informed decisions regarding antibiotic therapy. Over the past few decades, substantial advancements have been made in PK/PD research, leading to the identification of several key indicators that are essential for optimizing treatment outcomes while minimizing adverse effects. These indicators are invaluable tools for clinicians in selecting the most appropriate dose and dosing regimen of antibacterial agents. Advancements in experimental techniques, particularly the use of *in-vitro* PK models, have enabled researchers to replicate *in-vivo* conditions more accurately. Despite ongoing evolution in these methods, studies have demonstrated significant consistency in PK/PD indices and values across different animal models. This consistency indicates that, notwithstanding some inherent limitations, findings from animal studies can offer valuable insights into human responses.
